# A Strategy for Full Interrogation of Prognostic Gene Expression Patterns: Exploring the Biology of Diffuse Large B Cell Lymphoma

**DOI:** 10.1371/journal.pone.0022267

**Published:** 2011-08-04

**Authors:** Lisa M. Rimsza, Joseph M. Unger, Margaret E. Tome, Michael L. LeBlanc

**Affiliations:** 1 Department of Pathology, University of Arizona, Tucson, Arizona, United States of America; 2 Division of Public Health Sciences, Fred Hutchinson Cancer Research Center, Seattle, Washington, United States of America; University of Nebraska – Lincoln, United States of America

## Abstract

**Background:**

Gene expression profiling yields quantitative data on gene expression used to create prognostic models that accurately predict patient outcome in diffuse large B cell lymphoma (DLBCL). Often, data are analyzed with genes classified by whether they fall above or below the median expression level. We sought to determine whether examining multiple cut-points might be a more powerful technique to investigate the association of gene expression with outcome.

**Methodology/Principal Findings:**

We explored gene expression profiling data using variable cut-point analysis for 36 genes with reported prognostic value in DLBCL. We plotted two-group survival logrank test statistics against corresponding cut-points of the gene expression levels and smooth estimates of the hazard ratio of death versus gene expression levels. To facilitate comparisons we also standardized the expression of each of the genes by the fraction of patients that would be identified by any cut-point. A multiple comparison adjusted permutation p-value identified 3 different patterns of significance: 1) genes with significant cut-point points below the median, whose loss is associated with poor outcome (e.g. HLA-DR); 2) genes with significant cut-points above the median, whose over-expression is associated with poor outcome (e.g. CCND2); and 3) genes with significant cut-points on either side of the median, (e.g. extracellular molecules such as FN1).

**Conclusions/Significance:**

Variable cut-point analysis with permutation p-value calculation can be used to identify significant genes that would not otherwise be identified with median cut-points and may suggest biological patterns of gene effects.

## Introduction

Diffuse large B cell lymphoma (DLBCL) is an aggressive disease with a variable outcome. In order to quantify patient risk, numerous biomarkers have been identified that can be detected with a variety of methods. We recently described the use of a quantitative nuclease protection assay (qNPA) to measure gene expression levels from formalin fixed, paraffin embedded (FFPE) tissue blocks of DLBCL [1]. In a subsequent study of CHOP and rituximab-CHOP (R-CHOP) treated DLBCL cases, qNPA results for many genes were significantly associated with overall survival [2]. Initial data analysis was performed by categorizing patients into expression levels above and below the median level of expression. The best selected 2-variable model predicting overall survival in DLBCL was the combination of the major histocompatibility (MHC) class II antigen, HLA-DRB, and the cell cycle associated gene, MYC. In agreement with the literature, these results implicated lack of immunosurveillance and increased cell proliferation as important features that characterize the most aggressive B cell lymphomas [3]–[8].

We then further explored the relationship between expression levels and survival for these two genes. We plotted the score test statistic (logrank test statistic) from Cox regression for the association of gene expression quantile and survival, where gene expression was converted to a binary variable with cut-points defined along a continuous spectrum of low to high expression [9]. For HLA-DRB, the highest logrank statistic chi-square value indicating the most significant cut-point of gene expression was at the 20^th^ percentile. Many other cut-points were also significant [2]. This observation was in keeping with previous data demonstrating that there is a smooth non-linear association of MHC Class II expression levels as related to patient risk, with small incremental decreases in expression corresponding to increases in the hazard ratio of death with a sharp increase in hazard at lower levels of expression [10]. For MYC the most significant cut-point was at the 80^th^ percentile of expression. While the 80^th^ percentile was the optimal cut-point, there was a wide range of cut-point values that were also nominally significant [2]. This has biological implications for MYC, suggesting that incremental increases in MYC expression portend a worse prognosis with the sharpest increase in risk at higher levels of expression.

In the current study, we went on to perform this variable cut-point analysis on 36 genes to determine whether we could identify genes that might have significant cut-points other than the median and how that might be a factor in the reported discrepancies in prognostic value of genes by different investigators and techniques.

## Materials and Methods

### Ethics Statement

The project was approved by the University of Arizona Institutional Review Board (IRB) according to the principles expressed in the Declaration of Helsinki. The University of Arizona IRB specifically waived the need for informed consent for this project.

### Patient groups and mRNA data

We used the mRNA levels determined using qNPA (ArrayPlate^R^ Assay, High Throughput Genomics, Tucson, AZ) as described previously [1], [2]. Briefly, unstained FFPE sections of 209 DLBCL, previously treated with CHOP-like regimens (N = 93) or R-CHOP (N = 116) were subjected to the qNPA procedure. This process begins with cell lysis followed by exposure to specifically designed probe sets that bind to the target mRNA of interest. S1 nuclease is used to degrade all single stranded RNA and the surviving probes are identified by binding to linker probes and detection probes on the ArrayPlate^R^ followed by chemiluminescence and imaging. The study set of cases included FFPE blocks from cases of de novo, previously untreated DLBCL which had also been a part of 2 larger case series using gene expression profiling of snap frozen biopsies from patients treated with CHOP or R-CHOP and then later in a study of ArrayPlate^R^ gene expression technique on the corresponding FFPE blocks [2], [11], [12]. The customized ArrayPlate^R^ assay had been designed to assess the expression levels of 36 prognostic genes identified in DLBCL by different research groups and published in the literature. A list of the genes, their function (if known), and the reference from which they were chosen are listed in Table 1. All research was conducted under an IRB (human subjects committee) approved protocol from the University of Arizona. We obtained expression measurements with ≥95% success on all but 3 genes, and with ≤80% success on only one gene (HTR2B).

**Table 1:** Prognostic genes tested^1^.

Name in original reference	Alternative names	qNPA name	Reference	Function
BCL-6		BCL6*	Rosenwald 1/Lossos 6	Transcriptional repressor that controls germinal center formation [26], [27]
IMAGE 1334260	centerin/GCET1 (germinal center B-cell expressed transcript 1)	SERPINA9*	Rosenwald 2	Serpin (serine protease inhibitor) [28]
IMAGE 814622	GCET2 (germinal center B-cell expressed transcript 2)/HGAL (human germinal center-associated lymphoma)	GCET2	Rosenwald 3	Membrane-associated protein with a putative role in signal transduction [29]; myosin-interacting protein that is a putative inhibitor of cell migration [30]
HLA-DPa		HLA-DPA1	Rosenwald 4	Antigen presentation [31]
HLA-DQa		HLA-DQA1	Rosenwald 5	Antigen presentation [31]
HLA-DRa		HLA-DRA	Rosenwald 6	Antigen presentation [31]
HLA-DRb		HLA-DRB*	Rosenwald 7	Antigen presentation [31]
alpha-actinin		ACTN1*	Rosenwald 8	Non-muscle α-actinin isoform involved in bundling actin filaments and attaching them to focal adhesions; important for cell motility [32]
collagen type III alpha1		COL3A1*	Rosenwald 9	Type III fibrillar collagen; part of the extracellular matrix in lymph nodes [33], [34]
connective tissue growth factor		CTGF*	Rosenwald 10	Heparin and integrin binding protein involved in extracellular matrix remodeling [35]
fibronectin		FN1*	Rosenwald 11/Lossos 5	Extracellular integrin ligand involved in cell adhesion [36]
KIAA0233	Piezo1	FAM38A	Rosenwald 12	Multipass transmembrane protein involved in mechanotransduction and regulation of integrin activation [37], [38]
urokinase plasminogen activator	Urokinase/uPA	PLAU*	Rosenwald 13	Serine protease that activates plasminogen which results in extracellular matrix degradation [39]
C-MYC		MYC*	Rosenwald 14	Transcription factor that controls proliferation, growth, metabolism, microRNAs and apoptosis [40]
E21G3 Nucleostemin	NS	C20orf155	Rosenwald 15	Nucleolar GTP-binding protein that regulates cell cycle by regulating p53 and maintains nucleolar structure [41], [42]
NPM3	Nucleophosmin 3	NPM3	Rosenwald 16	Nucleolar protein that inhibits ribosome biogenesis and histone assembly and enhances transcription [43], [44]
BMP6	Bone morphogenetic protein-6	BMP6	Rosenwald 17	Cytokine that regulates B-cell lymphopoiesis [45]
LMO2	LIM domain only-2	LMO2	Lossos1	Transcription factor that regulates erythropoiesis and angiogenesis [46], [47]
BCL2		BCL2	Lossos 2	Membrane bound protein that prevents apoptosis [48]
SCYA3	MIP-1α(macrophage inflammatory protein-1)	CCL3	Lossos 3	Chemokine that recruits cells to sites of inflammation and inhibits hematopoietic stem cell proliferation [49]
CCND2	Cyclin D2	CCND2*	Lossos 4	Activator of cell cycle progression [50]
DRP2-dystrophin related protein 2		DRP2	Shipp 1	One of a class of structural proteins that maintains membrane–associated complexes at the points of intercellular contact [51]
PRKACB-protein kinase C beta 1	PKCβII	PRKCB1*	Shipp 2	Serine/threonine-specific kinase that plays a role in B-cell receptor signaling and B-cell development [52]
H731-nuclear antigen	Programmed Cell Death 4	PDCD4*	Shipp 3	Protein translation initiation factor inhibitor that is a putative context-specific tumor suppressor [53], [54]
3′ UTR of unknown protein	Microtubule-Associated Protein 1B	MAP1B	Shipp 4	Protein that stabilizes microtubules, attaches other proteins to microtubules and has a putative role in microvessicle trafficking [55], [56]
Transducin-like enhancer protein 1	Groucho	TLE1*	Shipp 5	Transcriptional co-repressor involved in differentiation of hematopoietic cells [57], [58]
Uncharacterized	citrin	SLC25A13	Shipp 6	Mitochondrial inner membrane aspartate-glutamate carrier that moves aspartate to the cytosol and NADH reducing equivalents into the mitochondria [59], [60]
PDE4B Phosphodiesterase 4B, cAMP-specific		PDE4B	Shipp 7	Phosphodiesterase that degrades cAMP to inactivate cAMP signaling [61]
Uncharacterized	UDP-Gal:betaGlcNAc β-1,4-galactosyltransferase polypeptide 1	B4GALT1	Shipp 8	Enzyme that transfers galactose to glycoproteins in a steriospecific manner; galactoproteins are involved in immune cell trafficking [62]
PRKCG Protein kinase C, gamma		PRKCG	Shipp 9	Serine/threonine–specific kinase activated by lipid signals and reactive oxygen species [63], [64]
Oviductal glycoprotein	MUC9	OVGP1	Shipp 10	Glycoprotein secreted by oviduct epithelial cells under estrogen control [65]
(MINO/NOR1) Mitogen induced nuclear orphan receptor		NR4A3	Shipp 11	Nuclear hormone receptor that regulates metabolism and inhibits leukemogenesis in a ligand-independent manner [66], [67]
Zinc-finger protein C2H2-150		ZNF212	Shipp 12	Putative transcription factor [68]
5-Hydroxytryptamine 2B receptor		HTR2B	Shipp 13	Serotonin receptor isotype involved in tumorigenesis [69], [70]
Catalase		CAT	Tome 1	Peroxisomal enzyme that metabolizes H_2_O_2_ [71]
Manganese superoxide dismutase		SOD2	Tome 2	Mitochondrial enzyme that metabolizes superoxide [72]

1 Last names with numbers refer to genes that are members of prognostic gene signatures previously reported in A. Rosenwald et al, I. Lossos et al, M. Shipp et al, and M. Tome et al. [12], [73], [74], [75].

### Variable cut-point and smooth hazard regression analysis

**Figure 1:**
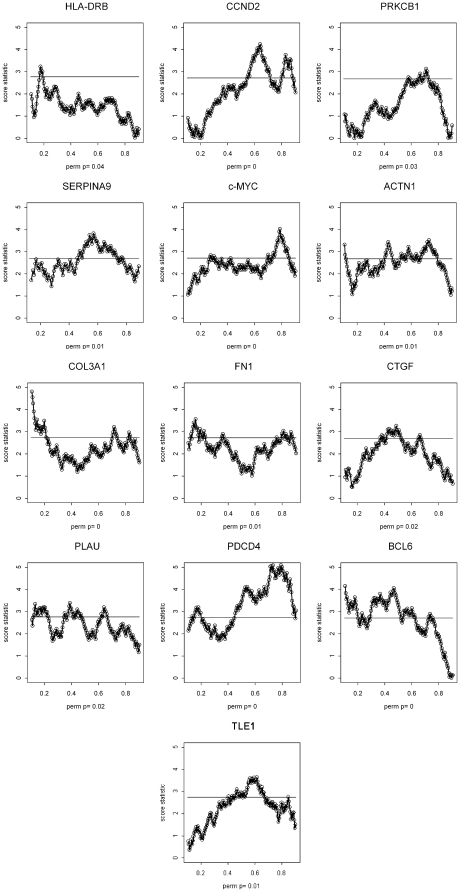
Graphs for each of the 13 genes with a significant logrank statistic (Z-value). On the Y-axis, an unadjusted score statistic of 2 corresponds to a p-value of approximately 0.05. On the X-axis, a value of 0.1 corresponds to the 10^th^ percentile of gene expression, 0.2 to the 20^th^ percentile, and etc up to the 90^th^ percentile of expression. Different cut-point values assessed for each gene are represented by the dots along the connected line of chi-square values. The solid horizontal line represents the 90^th^ percentile of the permutation distribution of the maximal score statistics. The range on the x-axis is from 10% to 90% of the distribution of the gene expression variable. An overall p-value adjusted for the permutation analysis is shown along the right sided Y-axis.

Variable cut-point (or split-point) analysis was performed on all 36 genes in order to discriminate between groups of patients with the most significant differences in overall survival. This statistical technique calculates the score test statistic from a Cox model (analogous to the logrank statistic) at a continuous spectrum of cut-points on the gene expression variable [13]. (Typically the maximum statistic is often used to define best split of patients.) In the plot (Figure 1), the vertical axis corresponds to the score statistic on the standard normal scale. To adjust for the evaluation of the large number of cut-point models, permutation sampling is used to control the family-wise type 1 error for each gene. The permutation p-values presented in the cut-point plots are based on 1000 samples, and the horizontal line on each plot corresponds to the 90^th^ percentile of the sampled permutation distribution of the maximum test statistic. Therefore, a cut-point statistical test reaching above the horizontal line has a permutation adjusted p-value of <0.10 [9]. Note that the 90^th^ percentile horizontal lines for the genes are at approximately 2.5 for most gene expression variables; if there were no adjustment for multiple comparisons, a value of 1.64 would correspond to a p-value of 0.1. Without this adjustment there would be the tendency to falsely believe moderately large test statistics correspond to real association, when observed associations could simply be due to the large number of cut-point models that have been investigated. In addition, to control statistical variability, a minimum possible subgroup size of 10% of total patients was set for each analysis. Since our previous test of panel-wide interaction between the CHOP and R-CHOP groups had shown no significance, we combined the 2 data sets for purposes of the current analysis [2]. However, the cut-point technique adjusted for treatment group (CHOP versus R-CHOP) as a main effect in the relative risk regression model, since R-CHOP is well known to be associated with improved survival. The cut-point technique also allows for more general adjustment of an existing prognostic model to assess the statistical significance of the addition of a new gene expression variable and cut-point. Analyses presented are based on overall survival, where overall survival is defined as the time from study registration until death. Patients without an observed death time are censored at the last known time under follow-up.

While the cut-point evaluation allows the assessment of statistical significance of multiple partitions of a gene expression variable, it does not directly lead to an estimate of the underlying regression function representing how gene expression is associated with survival. Therefore, we also used hazard regression modeling (based on a B-spline basis) to calculate smooth estimates of the hazard function for each gene [14]. An alternative estimation strategy for smooth hazard regression functions is by local likelihood [15]. In addition, we transformed each gene expression variable to be approximately uniformly distributed to make the analysis consistent with the cut-point analysis, which only depends on the rank of the gene expression variables. As done for the cut-point analysis, we adjusted for the two treatment groups (CHOP versus R-CHOP) via main effect in the relative risk regression model.

While our combination of cut-point analysis and smooth hazard regression modeling is useful for interpreting individual effects of a small set of continuous biological measurements, such as gene expression with censored survival patient outcome, there are other related statistical methods available for multivariable modeling and subgroup analysis. For instance, with respect to smooth regression modeling, there has been considerable study of generalized additive models, which consist of additive combinations of smooth univariate regression functions. For deriving subgroups in the context of many variables, the cut-point methods we proposed can be utilized recursively to cut-up or partition the data on multiple covariates to construct regression trees [16]. There is an extenstive discussion of other statistical or machine learning algorithms in Hastie et al. [17]. Due to the complexity of some of the multivariable models, their use is often better applied to patient prognostic predictions or subgroup stratification rather than probing the interpretation and clinical impact of individual gene expression measurements. In addition, alternatives to the smooth hazard regression models based on locally estimated quantiles of the survival distribution can be helpful for exploring gene effects [18]; however, we chose the hazard based methods for our exploration of DLBCL gene expression data given the relatively modest sample size. In addition, hazard regression methods tend to achieve better variance control in such cases.

## Results

We first generated a series of graphs for each of the 13 genes with significant logrank statistic (Z-value) (Fig. 1). The different cut-point values assessed for each gene are represented by the dots along the connected line of chi-square values. The solid horizontal line represents the 90^th^ percentile of the permutation distribution of the maximal score statistics under the assumption the gene is not associated with patient outcome (i.e., under the null hypothesis). Given the exploratory nature of this analysis, all values with a significance cut-off above the 90^th^ percentile line (type 1 error of 0.10) were considered significant. An overall p-value adjusted for the permutation analysis is presented on each of the panels. Note that only score statistics for cut-points that generate subgroups of patients with ≥10% of the sample size were considered, since smaller groups would probably not be considered useful clinically. We think it is useful to plot the cut-point analysis against the quantile of the gene expression distribution so that one could just read what fraction of the sample is above or below the cut-point.

Thirteen out of the 36 genes (36%) had at least 1 significant cut-point at p<0.10, including SERPINA9, HLA-DRB, ACTN1, COL3A, CTGF, FN1, PLAU, MYC, BCL6, CCND2, PRKCB1, PDCD4, and TLE1. Of these, 10 (77%) would have been significant at a pre-specified cut-point at the median (SERPINA9, ACTN1, COL3A, CTGF, PLAU, MYC, BCL6, CCND2, PDCD4, and TLE1) and 3 genes (or a relative 23% of the 13 genes) would not have been significant (HLA-DRB, FN1, and PRKCB1). Therefore, the median cut-point analysis would have missed detecting the significance of a notable selection of genes.

Inspection of the graphs revealed patterns that allowed us to classify the results into 3 different groups. The first group was defined as those genes with the significant cut-points only below the median. The second group was defined as genes with significant cut-points only above the median. The third group was defined as genes with significant cut-points above, below, or including the median.

The single gene in the first category was HLA-DRB, with the highest chi-square values all below the median and the most significant cut-point at the 20^th^ percentile. This pattern is consistent with a gene whose loss is associated with poor outcome.

The two genes that fell into the second category, showing significant cut-points above the median gene expression values, were CCND2 and PRKCB1. CCND2 is G1/S-specific regulator of cyclin-dependent kinases, and PRKCB1 functions as a serine- and threonine-specific protein kinase. This second pattern is consistent with genes whose over-expression is associated with poor outcome.

Ten genes fell into the third category, with significant cut-points above and below the median gene expression values. The genes in this category included ACTN1, COL3A, FN1, CTGF, PLAU, TLE1, PDCD4, MYC, SERPINA9, and BCL6. The first 5 of these 10 genes code for extra-cellular molecules. PDCD4 codes for an apoptosis related molecule, MYC is associated with proliferation and other cellular processes, while SERPINA9 and BCL6 are related to germinal center formation. While it wasn't explored in this analysis, an extended strategy for constructing prognostic groups of patients with significant cut-points at multiple points in the gene expression distribution (i.e., above and below the median) could be implemented. Here, a stage-wise approach would be appropriate. First, the maximal cut-point with all of the data would be identified; this defines two subgroups of patients. Next, evidence of a significant cut-point in either of the two remaining subgroups would be assessed. As before, permutation resampling methods would be used to determine evidence of further cut-points; this would indicate whether more than two prognostic groups, based on that gene, are needed.

**Figure 2:**
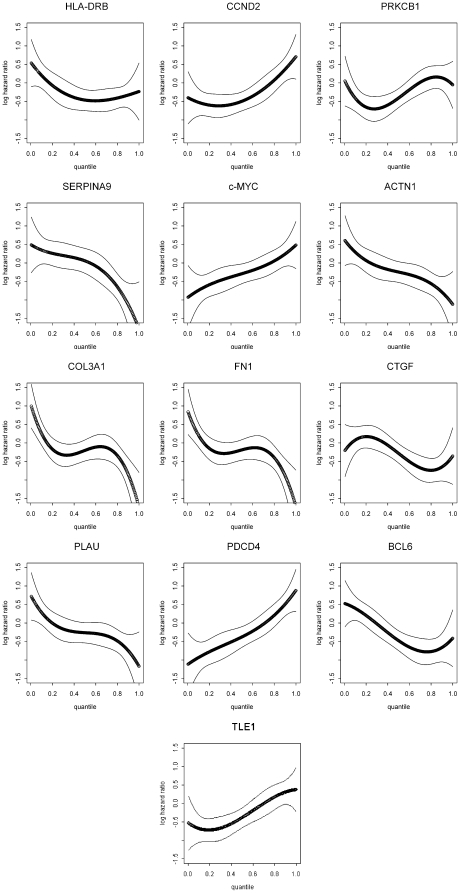
Hazard regression functions for the 13 genes with significant cut-points. The Y-axis shows the log of the hazard ratio of death. The X-axis shows the quantile of gene expression. The thin lines show the 90% confidence intervals.

Analysis of the cut-point graphs indicates whether or not expression of a particular gene is critical for patient outcome. However, to understand the impact of increasing or decreasing expression of a particular gene on patient outcome and gain insight into the tumor biology we generated hazard regression functions for the 13 genes with significant cut-points (Fig. 2). A hazard function that is increasing with respect to gene expression indicates a worse prognosis (or survival) with higher gene expression; conversely, a decreasing function implies improved survival for higher gene expression. The hazard regression functions confirm the importance of these genes and indicate whether an increase or decrease in expression is associated with better or worse patient survival. For example, examination of the hazard regression functions is in agreement with the known data on MYC. MYC over-expression in DLBCL results from translocations, increased gene copy number, or other mechanisms, and correlates with poor patient outcome [19]–[21].

In secondary analysis, we assessed whether adjustment for the International Prognostic Index (IPI) [22] mitigated the effect of gene expression on survival for the 13 genes described above. Results were similar, with ten of the 13 genes achieving family-wise error rate of <0.10.

## Discussion

While a large amount of effort in recent years has been devoted to evaluating thousands of genes from unfixed, snap frozen tissue, we have focused on a more detailed analysis of a smaller number of genes using FFPE. In this paper, we investigated the use of different cut-points for determining gene significance, which we applied here for the first time on GEP data for 36 genes on paraffin embedded tissue. We show that while using the median cut-point is often useful, the significance of some genes may be missed when the effect is limited to patients with only markedly high or low (rather than median) levels of expression.

Therefore, we believe the results more generally show that the variable cut-point method is a powerful tool to explore the relationship of gene expression data with outcomes. The strategy produces a sequence of decision rules to directly identify a group of patients, and hence, has a potential role in the translation of results to other studies. The second tool, smooth hazard regression, allows a finer understanding of the underlying biological relationships of gene expression with patient survival, but doesn't produce a decision rule. Therefore, this pair of tools together allows a fuller interrogation of gene expression data, an approach which has been largely overlooked under the current paradigm of performing simple univariate analyses at a genome-wide level. In practice, the choice of a cut-point derived by the methods we be propose can be used if there is not a specific cut-point of interest specified based on prior research. Our proposal would be to evaluate cut-points over a range of clinical interest. The choice of cut-point for subsequent clinical applications would often be the one that gave the largest test statistic value (or smallest p-value). However, one may choose other significant cut-point values that lead to larger subgroups depending on the clinical need in future studies. Importantly, given the multiple possible cut-points evaluated, the methodology includes an algorithm (permutation resampling) to control for potential false positive selection of a cut-point; that is, where there may not be a true association with patient outcome.

While we have focused our analysis and discussion on the understanding of individual genes, it is important to note that a cut-point algorithm can also be used to explore and draw inferences into whether or not other adaptively selected models might improve the existing prognostic models. Given a model with a set of specified variables and cut-points, the method allows one to statistically evaluate all cut-points over all remaining genes to see if any other variables would improve model performance. We assessed whether our prior model that included HLA-DRB and MYC could be improved by applying this method. We found that inclusion of the gene PDCD4, with a cut-point at the upper 27^th^ percentile of its distribution, had an adjusted p-value (controlling for multiple comparisons) of 0.001 to enter the model. Therefore, the 3-gene model including HLA-DRB, MYC, *and* PDCD4 appears to be preferred statistically over the prior 2-gene model. This improved model would likely not have been evident without using cut-point methodology.

In this project, a single median cut-point approach would have missed detecting a notable subset (23%) of the genes that were most significantly associated with survival at lower or higher expression cut-points. This may account for differences in significance of certain genes reported between different studies. Since a near complete loss of gene expression or high over-expression may be a relatively infrequent event for certain genes in some tumor types, these 2 categories of genes may be overlooked in general data analysis using median cut-points. We note that both in this data set and others, the statistically significant association of HLA-DR gene expression with survival would have been missed if only the median value of expression had been investigated.

Laboratory methods that either minimize or maximize signal will tend to underestimate the significance of genes with significant data cut-points at lower or higher levels of gene expression. For example, immunohistochemistry (IHC) often runs the chemical reaction through to equilibrium and may therefore over-estimate protein expression of genes by favoring a strong positive reaction. Furthermore, IHC is usually interpreted with simple descriptions of positive and negative staining based on visual inspection. Therefore, IHC strongly dichotomizes data and may miss the significance of lower or higher amounts of protein. Conversely methods that rely on high amounts of target for detection may also not reveal genes that are most significant at low levels of expression. It is therefore apparent that quantitative data with an appropriate dynamic range will be the most effective for exploring gene and protein expression patterns that play a prognostic role in DLBCL and other cancers. This factor might account for some of the discrepancies seen between gene expression and follow up confirmatory studies on their protein products.

By grouping similar hazard regression function patterns, we can speculate about the biological roles of the significant genes in DLBCL. These groups can differ somewhat from the categories generated in the cut-point analysis. Genes for which high expression is correlated with poor survival could be roughly described as oncogenes. MYC is a charter member of this category. Inspection of the MYC hazard regression function indicates that incremental increases have incremental effects on survival. This category would include CCND2, a protein closely related to proliferation, which has long been linked to outcome in DLBCL and mantle cell lymphomas [7], [12], [23]. PDCD4 also fits this pattern in DLBCL although studies in other cell types suggest PDCD4 can play a tumor suppressor role in other contexts [24].

Another hazard ratio pattern could be roughly described as genes for which loss of expression is associated with poor outcome. These genes have characteristics of tumor suppressor genes and include HLA-DRB. The pattern for HLA-DRB, which shows a sharp increase in hazard at lower levels of expression, also fits our previous data showing a loss of HLA-DRB is associated with poor outcome [2]. Previously, we had demonstrated an incrementally worse overall survival in patients as average major histocompatibility class II (MHC II) gene expression values (of which HLA-DRB is a principle gene) decreased by quantiles with the poorest outcome in patients at the 25^th^ percentile and below [10]. The current data also agree with our previous analysis that showed a non-linear association of HLA-DRA (part of the HLA-DR heterodimer) with patient hazard ratio of death - specifically with a sharp increase in hazard at lower levels of expression [10]. A comparison of the hazard regression functions for genes with a less well understood role in DLBCL to those of MYC and HLA-DRB provide insight as to their biological significance.

A third hazard ratio pattern is the genes with impact on survival especially at high and low expression. This pattern is most pronounced for COL3A1 and FN1, but PLAU also has this pattern. A gene expression pattern like this argues for threshold effects rather than a rheostat where incremental increases have incremental effects on survival. This type of pattern could reflect a requirement for other proteins in a complex to exert the full biological effect. Alternatively, this pattern could reflect a different impact of the gene in subgroups of DLBCL such as the cell of origin subtypes previously identified by GEP including germinal center B cell and activated B cell types [11], [12], [25]. The information from the hazard regression functions provides the basis for developing testable hypotheses to determine the importance of these genes for DLBCL biology.

In summary, we have demonstrated a method of statistical analysis that can be applied to GEP data and may reveal interesting associations with patient outcome. In particular, when data are evaluated by being split at expression levels other than the median, additional genes that correlate with patient outcome may be identified. A key component of the analysis is the use of the appropriate statistical techniques to control for false positive findings. To this end we have found re-sampling (in this study permutation sampling) to be extremely useful strategy to avoid over interpretation of flexible exploratory analysis such as cut-point techniques. Finally, while these genes and their cut-points will need to be validated in future studies, the results presented here may serve as hypothesis generating tools in regards to the use of particular genes at particular cut-points with possible implications for gene and tumor biology.

Software implementing the adjusted cut-point analysis is available from the final author.
